# Stochastic Periodic Solution of a Susceptible-Infective Epidemic Model in a Polluted Environment under Environmental Fluctuation

**DOI:** 10.1155/2018/7360685

**Published:** 2018-05-02

**Authors:** Yu Zhao, Jiangping Li, Xu Ma

**Affiliations:** ^1^School of Public Health and Management, Ningxia Medical University, Ningxia, Yinchuan 750004, China; ^2^School of Mathematics and Computer Science, Ningxia Normal University, Ningxia, Guyuan 756000, China

## Abstract

It is well known that the pollution and environmental fluctuations may seriously affect the outbreak of infectious diseases (e.g., measles). Therefore, understanding the association between the periodic outbreak of an infectious disease and noise and pollution still needs further development. Here we consider a stochastic susceptible-infective (SI) epidemic model in a polluted environment, which incorporates both environmental fluctuations as well as pollution. First, the existence of the global positive solution is discussed. Thereafter, the sufficient conditions for the nontrivial stochastic periodic solution and the boundary periodic solution of disease extinction are derived, respectively. Numerical simulation is also conducted in order to support the theoretical results. Our study shows that (i) large intensity noise may help the control of periodic outbreak of infectious disease; (ii) pollution may significantly affect the peak level of infective population and cause adverse health effects on the exposed population. These results can help increase the understanding of periodic outbreak patterns of infectious diseases.

## 1. Introduction

In Northern China, coal fire-power industries and heating systems, as well as vehicle emissions, all conduce to air pollution (airborne fine particulate matter PM_2.5_, PM_10_, and SO_2_, etc.), which has threatened the survival of exposed human population and affected the transmission of infectious diseases [1, 2]. Numerous studies have provided cumulative evidence of the health effects of particulate air pollution on the spread of infectious diseases (e.g., measles) [3, 4]. Therefore, investigating the role of pollution on the outbreak of infectious diseases is one of the most interesting and meaningful issues in the recent past [5].

**Figure 1:**
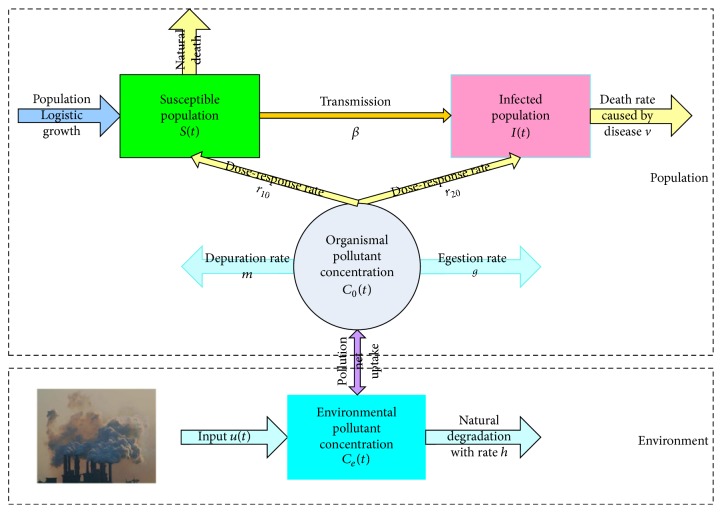
Flow chart of the interaction between environmental pollution and population, and the disease transmits from the susceptible subpopulation to infected subpopulation.

Dynamic mathematical models have provided a deeper understanding of the transmission process of infectious diseases [6, 7]. There are many interesting results (see, e.g., [8–10]), which show that the simple susceptible-infective (SI) model can fit the transmission process of some diseases (measles, chicken pox, etc.) well. Thus, based on the interaction between the environment and the population (see Figure 1), we incorporate the environmental pollution into the SI epidemic model. Let *S*(*t*), *I*(*t*), *C*_0_(*t*), and *C*_*e*_(*t*) denote the number of people in the susceptible population, the number of people in the infective population, and the concentration of pollution in the organism and in the environment at time *t*, respectively. The SI epidemic model in a polluted environment is as follows:

(1) dStdt=γSt1−St+ItK−βStIt−r10C0tSt−ξSt,dItdt=βStIt−r20C0tIt−vIt,dC0tdt=αCet−g+mC0t,dCetdt=ut−hCet;

all of the parameters are positive constants and the corresponding biological meanings are listed in Table 1. Liu et al. [8] obtained the sufficient conditions of the ultimate boundedness of solutions and the global asymptotical stability of the equilibria.

In real situations, as was pointed by Britton et al. [11], the transmission of infectious diseases is inevitably disrupted by unpredictable environmental conditions (e.g., absolute humidity [12], temperature [13]) making it more appropriate to use a stochastic model for biological parameters. For example, Yang et al. [14] observed the nonlinear effects of temperature and relative humidity on the incidence of measles. Thus, it is reasonable to model the environmental fluctuation as a stochastic transmission coefficient [15, 16]. If the transmission parameter *β* in the model (1) is subjected to some random environmental effects (temperature, humidity, etc.), then it is natural to consider that the transmission rate *β* is replaced by a random variable:

(2) $$\begin{array}{c} \tilde{\beta }=\beta +\sigma \varsigma \left(\;t\;\right), \end{array}$$

where *ς*(*t*) is the Gaussian white noise with mean zero and variance one, *ς*(*t*) = *dB*(*t*)/*dt*, and *B*(*t*) is a scalar Wiener Process defined in (*Ω*, *ℱ*, {*ℱ*_*t*_}_*t*≥0_, *P*), which is a complete probability space with a filtration {*ℱ*_*t*_}_*t*≥0_ satisfying the usual conditions (i.e., it is right continuous and *ℱ*_0_ contains all P-null sets). *σ* is the intensity of the white noise. Thus, we can incorporate the environmental white noises into model (3):

(3) dSt=St·γ1−St+ItK−βIt−r10C0t−ξdt−σStItdBt,dIt=ItβSt−r20C0t−vdt+σSt·ItdBt,dC0tdt=αCet−g+mC0t,dCetdt=ut−hCet.

Moreover, outbreaks of infectious diseases always fluctuate over time and exhibit seasonal patterns of incidence [17]. Ferrari et al. [18] pointed out that outbreaks of measles in the tropics have more variable seasonal patterns driven by accumulation and decline of susceptible individuals. Regular oscillatory patterns of measles outbreaks in Baltimore (USA) with an average period of three years have also been reported [19]. To describe the seasonal effect in the model, many existing studies [20–23] assume that the system parameters are subjected to a periodic rhythm. Therefore, we can further consider the following nonautonomous stochastic SI epidemic model as follows:

(4) dSt=Stγt1−St+ItKt−βtIt−r10tC0t−ξtdt−σtStItdBt,dIt=ItβtSt−r20tC0t−vtdt+σtStItdBt,dC0tdt=αCet−g+mC0t,dCetdt=ut−hCet,

with initial data

(5) S0=S0≥0,I0=I0≥0,0≤C00≤1,0≤Ce0≤1,

where *γ*(*t*), *K*(*t*), *β*(*t*), *r*_10_(*t*), *r*_20_(*t*), *ξ*(*t*), *σ*(*t*) are all positive, bounded, continuous *θ*-periodic functions.

Considering the periodic variation and pollution exposure of epidemic models and exploring the existence of stochastic periodic solutions are meaningful to predict and control the outbreaks of infectious diseases. Such analysis has benefited from the theoretical contributions about the nonautonomous stochastic system [24, 25]. We also see that there has been some research in this respect [20, 21, 26, 27]. For example, Jiang et al. [21] considered a stochastic nonautonomous competitive Lotka-Volterra model in a polluted environment and then derived sufficient criteria for the existence and global attractivity of a nontrivial positive periodic solution. Xie et al. [27] presented a stochastic hepatitis B virus infection model with logistic hepatocyte growth and showed that the model has at least one periodic solution. More related results can be found in [22, 28, 29]. To the best of our knowledge, there are few results about the periodic solution of a stochastic SI epidemic model in a polluted environment. Therefore, the main objective of this paper is to concentrate on the effects of pollution and environmental fluctuation on the existence of the positive periodic solution.

The rest of this paper is organized as follows: in the next Section 2, we present the underlying mathematical analysis: the existence of the global positive solution, the sufficient conditions for the nontrivial stochastic periodic solution, and the boundary periodic solution of disease extinction are derived. The subsequent Section 3 describes the numerical simulation, based on the case of measles, carried out to support the theoretical results. Finally, in the last Section 4, the conclusion is presented.

## 2. Mathematical Analysis

### 2.1. Preliminary

Since *C*_0_(*t*), *C*_*e*_(*t*) are the concentrations of the pollution and 0 ≤ *C*_0_(*t*) < 1 and 0 ≤ *C*_*e*_(*t*) < 1 must be satisfied, we assume the following.

**Assumption 1 (0 < *α* ≤ *g* + *m*, *u*(*t*) ≤ *h*).**

Notice that *u*(*t*) is a positive *θ*-periodic continuous function, so we can prove the following.

**Lemma 2 .**

For model (4), we have lim_*t*→*∞*_|*C*_0_(*t*) − *C*_0_^*∗*^(*t*)| = 0; here

(6) $$\begin{array}{c} C_{0}^{*}\left(\;t\;\right)=\frac{\int_{t}^{t+\theta }e^{\left(g+m\right)\left(s-t\right)}\alpha C_{e}\left(s\right)ds}{e^{\left(g+m\right)\theta }-1}. \end{array}$$

The proof of this lemma is provided in Appendix.

From now on, we will only consider the following system:

(7) dSt=Stγt1−St+ItKt−βtIt−r10tC0∗t−ξtdt−σtStItdBt,dIt=ItβtSt−r20tC0∗t−vtdt+σtStItdBt.

For a bounded function on [0, *∞*, say, *f*(*t*), define

(8) fu=supt∈0,∞⁡ft,fl=inft∈0,∞⁡ft,fθ=1θ∫0θfsds.

Now, we shall give some definitions and Lemmas with respect to the periodic Markov process *X*(*t*) as the solution of stochastic system

(9) Xt=Xt0+∫t0tbs,Xsds+∑r=1k∫t0tσrs,XsdBrs,x∈Rl.

**Definition 3 (see [24]).**

A stochastic process *ξ*(*t*) = *ξ*(*t*, *ω*)  (−*∞* < *t* < *∞*) is said to be periodic with period *θ*, if for every finite sequence of numbers *t*_1_, *t*_2_,…, *t*_*n*_ the joint distribution of the random variables *ξ*(*t*_1_ + *h*),…, *ξ*(*t*_*n*_ + *h*) is independent of *h*, where *h* = *kθ*, *k* = ±1, ±2,….

**Remark 4 .**

It follows from [24] that a stochastic Markov process *X*(*t*) is *θ*-periodic if and only if its transition probability function is *θ*-periodic and the function *𝒫*_0_(*t*, *A*) = *𝒫*{*X*(*t*) ∈ *A*} satisfies

(10) P0s,A=∫RlP0s,dxPs,x,s+θ,A≡P0s+θ,A,

for every *A* ∈ *𝔅*, where *𝔅* denotes the Borel *σ*-algebra in *ℝ*^*l*^.

Let *ℒ* be a linear operator defined by

(11) L=∂∂t+∑i=1nbit,x∂∂xi+12∑i,j=1naij∂2∂xi∂xj,aij=∑r=1kσrit,xσrjt,x.

**Lemma 5 (see [24]).**

Suppose that the coefficients of system (13) are *θ*-periodic in *t* and satisfy

(12) bs,x−bs,y+∑r=1kσrs,x−σrs,x≤Cx−y,bs,x+∑r=1kσrs,x≤C1+x,

in every cylinder *I* × *U*, where *C* is a constant; and suppose further that there exists a function *V*(*t*, *x*) ∈ *ℝ*^*l*^ which is *θ*-periodic in *t*, satisfying

(13) infx>R⁡Vt,x⟶∞,as  R⟶∞,

(14) LVt,x≤−1,outside  some  compact  set.

Then, there exists a solution of system (9) which is a *θ*-periodic Markov process.

**Remark 6 .**

According to the proof of Lemma 2.1 in [24], condition (14) is only used to guarantee the existence and uniqueness of the solution of system (9).

Next, we have the following theorem.

**Theorem 7 .**

For any given initial value (5), model (7) has a unique positive solution (*S*(*t*), *I*(*t*)) on *t* ≥ 0, and the solution will remain in *ℝ*_+_^2^ with probability one.

The proof of this Theorem is provided in Appendix.

### 2.2. Existence of the Positive Stochastic Periodic Solution

In this section, we shall prove the existence of a positive stochastic periodic solution of models (4) and (7). Firstly, we define

(15) λθ=1θ∫0θγs−r10s+r20sC0∗s−12γuKuγl2σ2s−ξs−vsds.

Now, we obtain the following result regarding the existence of a positive periodic solution of model (7).

**Theorem 8 .**

If *λ*_*θ*_ > 0, then model (7) at least has one positive *θ*-periodic solution (*S*^*∗*^(*t*), *I*^*∗*^(*t*)).

**Proof**

To prove the existence of a positive *θ*-periodic solution of model (7), it follows from Lemma 2 and Remark 6 that we need to find a *C*^2^-function *V*(*t*, *S*, *I*) and a closed set Θ ∈ *ℝ*_+_^2^ such that (13) and (14) hold. Firstly, we assume that *γ*^*u*^ + *γ*^*u*^/*K*^*l*^ − *β*^*l*^ − (*r*_10_*C*_0_^*∗*^ + *ξ*)^*l*^ > 0 and define a nonnegative function as follows:

(16) $$\begin{array}{c} V\left(\;t,S,I\;\right)=V_{1}\left(\;t,S\;\right)+V_{2}\left(\;t,S,I\;\right)+V_{3}\left(\;t\;\right), \end{array}$$

where

(17) V1t,S=−Mln⁡St−Mln⁡It,V2t,S,I=12St+It2+MSt+It,V3t=Mω¯t,M=2λθmax⁡1,supS,I∈R+2⁡−γlKuS3+γu−r10C0∗+ξl−MγlKu+σu2I2·S2+γu−r20C0∗+vl−r10C0∗+ξl−MγlKuSI−r20C0∗+vlI2+MγuKl+γu−βl−r10C0∗+ξlS+MγlKuS3+γu−r10C0∗+ξl−MγlKu+σu2I2γuKl+βu−r20C0∗+vlI.

It is easy to see that *Mλ*_*θ*_/2 ≥ 1 and $\bar{\omega }(t)$ satisfies the following:

(18) ω¯˙t=−λθ+γt−r10t+r20tC0∗t−12γuKuγl2σ2t−ξt−vt,ω¯0=0.

Integrating (18) from *t* to *t* + *θ* yields

(19) ω¯t+θ−ω¯t=∫tt+θω¯˙sds=∫tt+θ−λθ+γs−r10s+r20sC0∗s−12γuKuγl2σ2s−ξs−vsds−∫0θ−λθ+γs−r10s+r20sC0∗s−12γuKuγl2σ2s−ξs−vsds=0.

Thus, we can see that $\bar{\omega }(t)$ is a *θ*-periodic function on [0, *∞* and

(20) $$\begin{array}{c} \underset{\epsilon \to 0,\left(S,I\right)\in R_{+}^{2}\setminus \Theta_{\epsilon }}{\mathrm{liminf}}V\left(\;t,S,I\;\right)=\infty , \end{array}$$

where Θ_*ϵ*_ = {(*S*, *I*):(*S*, *I*)∈(*ϵ*, 1/*ϵ*)×(*ϵ*, 1/*ϵ*)}. Thus, *V*(*t*, *S*, *I*) is *θ*-periodic with respect to *t*.

Now, we have the requisite information to verify (14) in Lemma 5. Applying the Itô formula to *V*(*t*, *S*, *I*), one can obtain

(21) LV1=−Mγt−γtktS+I−r10tC0∗t−βtI−ξt−σ2tI22+βtS−r20tC0∗t−vt−σ2tS22≤M−γt−r10t+r20tC0∗t−ξt−vt+γuKlS+γuKl+βuI+γuKuγl2σ2t−βlS=M−γt−r10t+r20tC0∗t−12γuKuγl2σ2t−ξt−vt+γuKlS+γuKl+βuI−βlS,LV2=S+IγtS1−S+IKt−r10tC0∗t+ξtS−r20tC0∗t+vtI+Mγt·S1−S+IKt−r10tC0∗t+ξtS−r20tC0∗t+vtI+σ2tS2I2≤γuS2−γlKuS3−γlKuS2I−r10C0∗+ξlS2−r20C0∗+vl·SI−γlKuS2I−γlKuSI2−r10C0∗+ξlSI−r20C0∗+vlI2+γuSI+σu2S2I2+MγuS−MγlKuS2−MγlKuIS−Mr10C0∗+ξlS−Mr20C0∗+vlI≤−γlKuS3+γu−r10C0∗+ξl−MγlKu+σu2I2S2−r20C0∗+vlI2+γu−r20C0∗+vl−r10C0∗+ξl−MγlKuSI+Mγu−r10C0∗+ξlS−Mr20C0∗+vlI.

From (18) and (21), we can get that

(22) LV=−Mλθ+MγuKl−βlS+MγuKl+βuI−γlKuS3+γu−r10C0∗+ξl−MγlKu+σu2I2S2−r20C0∗+vlI2+γu−r20C0∗+vl−r10C0∗+ξl−MγlKuSI+Mγu−r10C0∗+ξlS−Mr20C0∗+vlI.

Define a closed set

(23) $$\begin{array}{c} \Theta_{\epsilon }=\left\{\;\left(\;S,I\;\right)\in R_{+}^{2}:\epsilon \leq S\leq \frac{1}{\epsilon },\;\;\epsilon \leq I\leq \frac{1}{\epsilon }\;\right\}, \end{array}$$

where 0 < *ϵ* < 1 is a sufficiently small number such that

(24) −Mλθ+MγuKl+γu−βl−r10C0∗+ξlϵ+C1≤−1,

(25) −Mλθ−Mβl+r10C0∗+ξl1ϵ+C2≤−1,

(26) −Mλθ−r20C0∗+vl1ϵ+C3≤−1,

where *C*_*i*_, *i* = 1,2, 3 are positive constants defined in (29), (32), and (34) later. Moreover, we denote

(27) Θϵ1=S,I∈R+2:0<S<ϵ,Θϵ2=S,I∈R+2:0<I<ϵ,Θϵ3=S,I∈R+2:S>1ϵ,Θϵ4=S,I∈R+2:I>1ϵ.

Then Θ_*ϵ*_^*c*^ = *ℝ*_+_^2^∖Θ_*ϵ*_ = Θ_*ϵ*_^1^ ∪ Θ_*ϵ*_^2^ ∪ Θ_*ϵ*_^3^ ∪ Θ_*ϵ*_^4^. Next, we shall prove *ℒV*(*t*, *S*, *I*)≤−1 on [0, *∞*] × Θ_*ϵ*_^*c*^.

*Case 1. *On Θ_*ϵ*_^1^, we have 0 < *S* < *ϵ*:

(28) LV=−Mλθ+MγuKl+γu−βl−r10C0∗+ξlS−γlKuS3+γu−r10C0∗+ξl−MγlKu+σu2I2S2−r20C0∗+vlI2+γu−r20C0∗+vl−r10C0∗+ξl−MγlKuSI+MγuKl+βu−r20C0∗+vlI≤−Mλθ+MγuKl−βl+γu−r10C0∗+ξlϵ+C1,

where

(29) C1=supS,I∈R+2⁡−γlKuS3+γu−r10C0∗+ξl−MγlKu+σu2I2S2−r20C0∗+vlI2+γu−r20C0∗+vl−r10C0∗+ξl−MγlKuSI−Mr20C0∗+vl−γuKl−βuI.

Therefore, we can say that *ℒV*(*t*, *S*, *I*)≤−1 on [0, *∞*] × Θ_*ϵ*_^1^ in lieu of (24).

*Case 2. *On Θ_*ϵ*_^2^, we have 0 < *I* < *ϵ*:

(30) LV≤−Mλθ2+−Mλθ2+supS,I∈R+2−γlKuS3+γu−r10C0∗+ξl−MγlKu+σu2I2S2−r20C0∗+vlI2+γu−r20C0∗+vl−r10C0∗+ξl−MγlKuSI+MγuKl+γu−βl−r10C0∗+ξlS+MγuKl+βu−r20C0∗+vlI.

It follows from the definition of *M* that *ℒV* ≤ −*Mλ*_*θ*_/2 ≤ −1, which implies *ℒV*(*t*, *S*, *I*)≤−1 on [0, *∞*] × Θ_*ϵ*_^2^.

*Case 3. *On Θ_*ϵ*_^3^, we have *S* > 1/*ϵ*:

(31) LV=−Mλθ−Mβl+r10C0∗+ξlS+−γlKuS3+γu−r10C0∗+ξl−MγlKu+σu2I2S2−r20C0∗+vlI2+γu−r20C0∗+vl−r10C0∗+ξl−MγlKuSI+MγuKl+βu−r20C0∗+vlI+MγuKl+γuS≤−Mλθ−Mβl+r10C0∗+ξl1ϵ+C2,

where

(32) C2=supS,I∈R+2⁡−γlKuS3+γu−r10C0∗+ξl−MγlKu+σu2I2S2−r20C0∗+vlI2+γu−r20C0∗+vl−r10C0∗+ξl−MγlKuSI+MγuKl+βu−r20C0∗+vlI+MγuKl+γuS.

According to (25), one can get that *ℒV*(*t*, *S*, *I*)≤−1 on [0, *∞*] × Θ_*ϵ*_^3^.

*Case 4. *On Θ_*ϵ*_^4^, we have *I* > 1/*ϵ*:

(33) LV=−Mλθ−r20C0∗+vlI+−γlKuS3+γu−r10C0∗+ξl−MγlKu+σu2I2S2−r20C0∗+vlI2+γu−r20C0∗+vl−r10C0∗+ξl−MγlKuSI+MγuKl+βuI+MγuKl+γu−βl−r10C0∗+ξlS≤−Mλθ−r20C0∗+vl1ϵ+C3,

where

(34) C3=supS,I∈R+2⁡−γlKuS3+γu−r10C0∗+ξl−MγlKu+σu2I2S2−r20C0∗+vlI2+γu−r20C0∗+vl−r10C0∗+ξl−MγlKuSI+MγuKl+βuI+MγuKl+γu−βl−r10C0∗+ξlS.

In view of (26), we can deduce that *ℒV*(*t*, *S*, *I*)≤−1 on [0, *∞*] × Θ_*ϵ*_^4^.

In summary, we can draw the conclusion that

(35) $$\begin{array}{c} LV\left(\;t,S,I\;\right)\leq -1\;\text{for every }\left[0,\infty \right]\times \Theta_{\epsilon }^{c}. \end{array}$$

Thus, the condition (14) of Lemma 5 is satisfied. Consequently, model (7) at least has one positive stochastic *θ*-periodic solution.

**Remark 9 .**

Theorem 8 implies that the intrinsic growth rate of population should overcome the extinction risks of infected disease and pollution in order to guarantee the survival of the population. In addition, the condition *λ*_*θ*_ means that the susceptible population evolution dynamic (*γ*, *K*), the dose-response rates (*r*_*i*0_, *i* = 1,2), and intensity of noise (*σ*) play an important role in determining the periodic outbreak of infectious disease; that is, reducing the possibility of *λ*_*θ*_ > 1 is beneficial to the control of the periodic outbreak of infectious diseases.

**Remark 10 .**

In contrast to the authors in [8] assume that the exogenous rate of pollutant input *u*(*t*) has constant limit(i.e., lim_*t*→*∞*_*u*(*t*) = *u*^*∗*^) and derived the stability of equilibria, which ignored the periodicity of model. In this paper, we consider the limit periodic system with attractiveness (i.e., lim_*t*→*∞*_|*C*_0_(*t*) − *C*_0_^*∗*^(*t*)| = 0) and obtain the positive stochastic periodic solution, which extends the results in [8] to a stochastic nonautonomous situation.

Combining Lemma 2 and Theorem 8, we can have the following result on the existence of positive stochastic periodic solution of model (4).

**Theorem 11 .**

Under the conditions of Theorem 8, model (4) at least has one positive stochastic *θ*-periodic solution (*S*^*∗*^(*t*), *I*^*∗*^(*t*), *C*_0_^*∗*^(*t*), *C*_*e*_^*∗*^(*t*)).

### 2.3. The Boundary Periodic Solution of Disease Extinction

In this section, we shall obtain the sufficient conditions for disease extinction. Firstly, we define

(36) $$\begin{array}{c} \lambda_{\theta }^{0}=\frac{1}{\theta }\overset{\theta }{\underset{0}{\int }}\left[\;\frac{\beta^{2}\left(s\right)}{2\sigma^{2}\left(s\right)}-r_{20}\left(\;s\;\right)C_{0}^{*}\left(\;s\;\right)-v\left(\;s\;\right)\;\right]ds. \end{array}$$

Next, we prove the following theorem.

**Theorem 12 .**

For model (7), if *λ*_*θ*_^0^ < 0, then the disease *I*(*t*) goes to extinct almost surely.

**Proof**

Define *W*(*I*) = ln⁡*I*(*t*) for *I* ∈ [0, *γ*^*u*^*K*^*u*^/*γ*^*l*^]. Utilizing the Itô formula to model (7) yields

(37) $$\begin{array}{c} dW\left(\;I\left(\;t\;\right)\;\right)=LW\left(\;I\left(\;t\;\right)\;\right)dt+\sigma \left(\;t\;\right)S\left(\;t\;\right)dB\left(t\right), \end{array}$$

where

(38) LWIt=βtSt−r20tC0∗t−vt−σ2t2S2t≤β2t2σ2t−r20tC0∗t−vt.

Substituting (38) into (37) and integrating both sides of (37), one can deduce that

(39) 1t∫0tln⁡ItI0≤1t∫0tβ2s2σ2s−r20sC0∗s−vsds+Mtt,

where *M*(*t*) = ∫_0_^*t*^*σ*(*s*)*S*(*s*)*dB*(*s*) is a martingale with the following quadratic variation:

(40) Mt,Mtt=∫0tσuSu2du≤sups≥0⁡σuγuKuγl2,a.s.

According to the strong law of large numbers for martingales [32], we can get

(41) $$\begin{array}{c} \underset{t\to \infty }{lim}\frac{M\left(t\right)}{t}=0,\;\text{a.s.} \end{array}$$

Combining (39), (41), and the coefficients' periodicity of model (7), we get

(42) limt→∞1t∫0tln⁡ItI0≤limt→∞⁡1t∫0tβ2s2σ2s−r20sC0∗s−vsds+limt→∞⁡Mtt=1θ∫0θβ2s2σ2s−r20sC0∗s−vsds=λθ0<0,

and hence lim_*t*→*∞*_*I*(*t*) = 0, a.s.

Note the fact that when lim_*t*→*∞*_*I*(*t*) = 0, model (7) reduces to the following nonautonomous system:

(43) $$\begin{array}{c} d\left(\;S\left(\;t\;\right)\;\right)=S\left(\;t\;\right)\left[\;a\left(\;t\;\right)-b\left(\;t\;\right)S\left(\;t\;\right)\;\right], \end{array}$$

where *a*(*t*) = *γ*(*t*) − *r*_10_(*t*)*C*_0_^*∗*^(*t*) − *ξ*(*t*), *b*(*t*) = *γ*(*t*)/*K*(*t*) are all *θ*-periodic functions. Define

(44) $$\begin{array}{c} \lambda_{\theta }^{S}=\frac{1}{\theta }\overset{\theta }{\underset{0}{\int }}\left[\;\gamma \left(\;s\;\right)-r_{10}\left(\;s\;\right)C_{0}^{*}\left(\;s\;\right)-\xi \left(\;s\;\right)\;\right]ds. \end{array}$$

Therefore, we have the following *θ*-periodic solution result of system (43).

**Lemma 13 (see Globalism [33]).**

For system (43), if *λ*_*θ*_^*S*^ > 0 then it has a stable positive *θ*-periodic solution *S*_*θ*_^0^(*t*) which satisfies

(45) $$\begin{array}{c} \frac{1}{S_{\theta }^{0}\left(t\right)}=\frac{\int_{t}^{t+\theta }\exp\left\{\int_{t}^{s}a\left(\tau \right)d\tau \right\}b\left(s\right)ds}{\exp\left\{\int_{0}^{\theta }a\left(\tau \right)d\tau \right\}-1},\;t\geq 0. \end{array}$$

In summary, we obtain the following.

**Theorem 14 .**

For model (7), if *λ*_*θ*_^0^ < 0 and *λ*_*θ*_^*S*^ > 0, then it has a boundary periodic solution of disease extinction (*S*_*θ*_^0^(*t*), 0).

**Remark 15 .**

The transmission coefficient *β*, intensity of noise *σ*, and pollution level *r*_20_*C*_0_^*∗*^ may determine the fate of the evolution of infected population.

## 3. Numerical Simulation

In this section, we shall verify the above theoretical results and illustrate the effects of environmental fluctuation and pollution on the periodic outbreak of infectious disease. With help from MATLAB (Mathworks, Inc., Natick, MA, USA) and Milsteins higher order method [34], which is a powerful tool for solving stochastic differential equations, we consider the following discretized equation of model (7) at *t* = (*k* + 1)Δ*t*, *k* = 0,1,…:

(46) Sk+1=Sk+SkγkΔt1−Sk+IkKkΔt−βkΔtIk−r10kΔtC0∗kΔt−ξkΔtΔt−SkIkσkΔtkΔtξk+σ2kΔt2ξk2Δt−Δt,Ik+1=Ik+IkβkΔtSk−r20kΔtC0∗kΔt−vkΔtΔt+SkIkσkΔtkΔtξk+σ2kΔt2ξk2Δt−Δt,

where *ξ*_*k*_ are the *N*(0,1)-distribution independent Gaussian random variables. Let us assume that

(47) γt=3.5+0.5cos⁡t12,Kt=100+5cos⁡t12,ξt=0.34+0.05cos⁡t12,vt=0.05+0.01cos⁡t12,C0∗t=0.5+0.05cos⁡t12,σt=0.01+0.005cos⁡t12,βt=0.25+0.05cos⁡t12.

**Example 16 .**

To illustrate the effect of pollution on the periodic outbreaks of infectious disease, we look at the following two cases that differ with respect to the average pollution level.

*Case (i)*. Assume that *r*_10_(*t*) = 0.15 + 0.05cos(*t*/12), *r*_20_(*t*) = 0.2 + 0.05cos(*t*/12). After some simple calculations, we can see that *γ*^*u*^ + *γ*^*u*^/*K*^*l*^ − *β*^*l*^ − (*r*_10_*C*_0_^*∗*^ + *ξ*)^*l*^ = 1.8711 > 0 and

(48) $$\begin{array}{c} \lambda_{\theta }=3.3871>0. \end{array}$$

**Figure 2:**
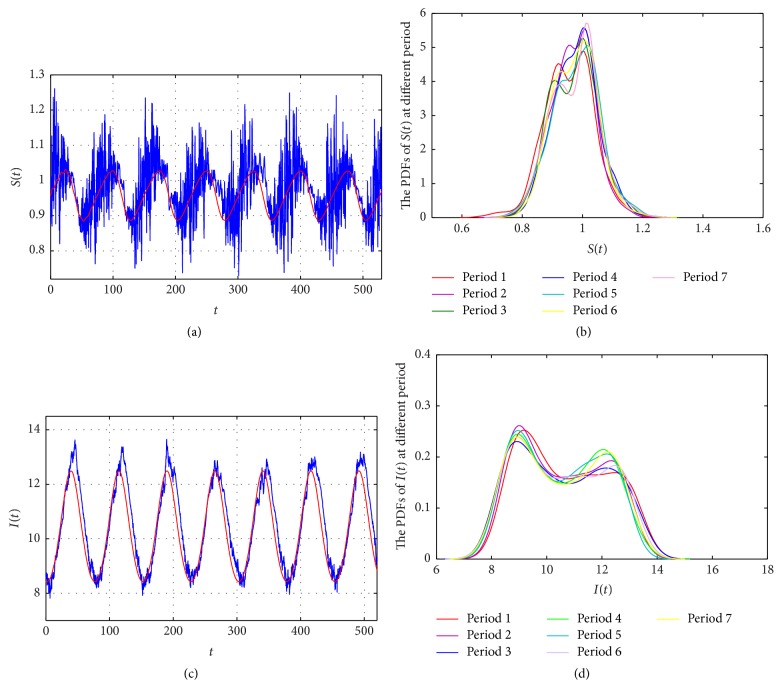
(a), (c) The sample trajectory of *S*(*t*) and *I*(*t*) of model (7) (blue lines) and their corresponding deterministic periodic solution (red lines), respectively. (b), (d) the probability density functions (PDFs) of *S*(*t*) and *I*(*t*) of model (7) in different periods, respectively. The initial value is (1,11) and the parameter values are used as in Example 16.

Thus, it follows from Theorem 8 that model (7) has at least one positive 24*π*-periodic solution. As shown in Figures 2(a) and 2(c), the probability density functions (PDFs) of (*S*(*t*), *I*(*t*)) of model (7) are nearly equal (from the shape of stationary distribution aspect) to each other in different periods, which supports the definition of a periodic Markov process (see Figures 2(b) and 2(d)). Therefore, the solution process of model (7) is a 24*π*-periodic Markov process. Additionally, in the absence of white noise, model (7) reduces to a deterministic system. Hence, we also plot the trajectories of the corresponding deterministic model (7) in Figures 2(a) and 2(c) (red lines). In summary, it can be observed that the sample trajectories of the stochastic model (7) have regular periodicity under small environmental fluctuations, and fluctuation happens around the periodic solution of the corresponding deterministic counterparts.

**Figure 3:**
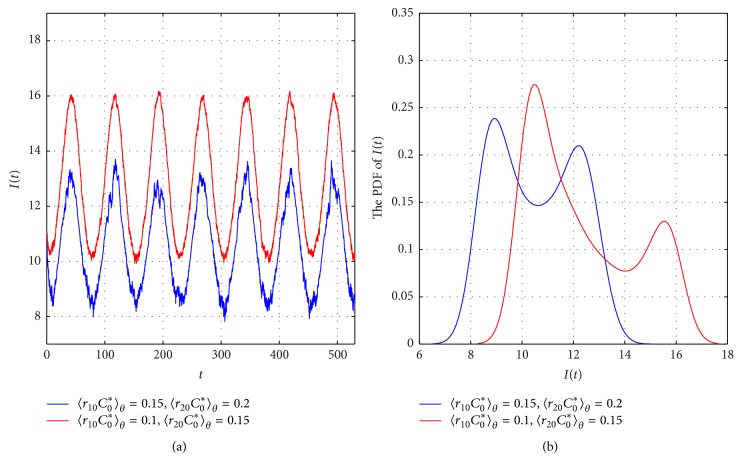
(a) The sample trajectory *I*(*t*) of model (7) corresponding to different average pollution level *r*_*i*0_*C*_0_^*∗*^, respectively. (b) the PDFs of *I*(*t*) of model (7) in the same period with different average pollution level, respectively.

*Case (ii)*. Assume that *r*_10_(*t*) = 0.1 + 0.05cos(*t*/12), *r*_20_(*t*) = 0.15 + 0.05cos(*t*/12). After some simple calculations, we can check that *γ*^*u*^ + *γ*^*u*^/*K*^*l*^ − *β*^*l*^ − (*r*_10_*C*_0_^*∗*^ + *ξ*)^*l*^ = 1.9711 > 0 and *λ*_*θ*_ = 3.4121 > 0. According to Theorem 8, model (7) has a stochastic 24*π*-periodic solution (see Figure 3(a), red lines). We can also observe from Figure 3(a) that due to the decrease in pollution, the peak level of infective population increases. Meanwhile, the corresponding PDF moves to the right position, which implies a higher number for population of *I*(*t*) (see Figure 2(b)). Thus, pollution may increase the peak level of infective population.

Next, we shall check the existence of a boundary periodic solution.

**Example 17 .**

Let us assume that *r*_10_(*t*) = 0.15 + 0.05cos(*t*/12), *r*_20_(*t*) = 0.2 + 0.05cos(*t*/12), *β*(*t*) = 0.0015 + 0.0005cos(*t*/12); then we can calculate that

(49) λθ0=−0.2388<0,λθS=3.011>0.

**Figure 4:**
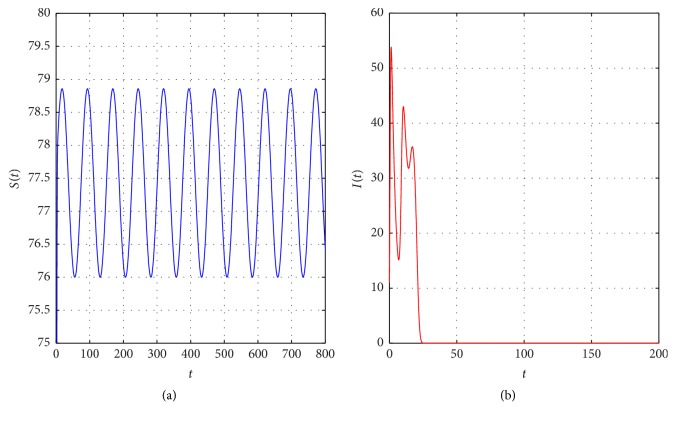
The sample trajectory of the boundary periodic solution (*S*_*θ*_^0^(*t*), 0) of model (7), with initial values (75,10).

It follows from Theorem 12 that there exists a boundary periodic solution of disease extinction (*S*_*θ*_^0^(*t*), 0) of model (7), which is consistent with the simulation results as shown in Figure 4.

Now, we are in a position to see the fit of model (7) for a real-world situation (the case of measles).

**Example 18 .**

Measles is a highly contagious airborne infectious disease caused by the measles virus, which spreads easily through coughing and sneezing of infected people. Major epidemics occur approximately every 2-3 years, causing an estimated 2.6 million deaths each year [31]. Recent research has showed that the incidence of measles is related to air pollution in China [1, 3, 35] and has provided cumulative evidence of the adverse health effects of particulate air pollution and dust. Thus, using measles as an example, we have the requisite information to fit a real-world situation such as the outbreak of measles by using model (7). The data source for the cases of measles was from the Chinese center for disease control and prevention (CCDCP) [36]. The parameters of the simulation are listed in Table 2.

**Figure 5:**
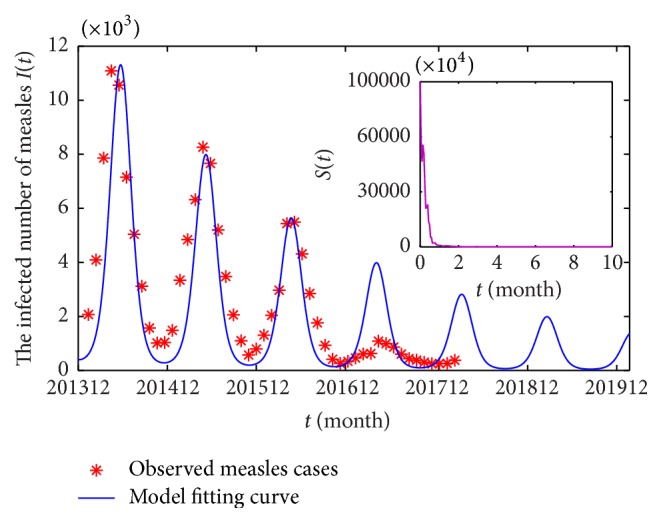
Observed (red double cross pattern) of cases of measles *I*(*t*) during Jan 2013 to Dec 2016 and model fitted and predicted cases of measles (blue line) from Jan 2013 to Dec 2019. The inner panel is the sample path of *S*(*t*).

It can be seen from Figure 5 that our simulation, based on model (7), is a good fit compared to the data on the observed cases of measles in the period from Jan 2014 to Dec 2016. However, the results of fitting the model in the period from Jan 2017 to Dec 2017 are not good; this may be due to the following reasons: (1) by 2016, the government and prevention departments push to improve vaccine coverage may have resulted in the drop in the cases of measles [31]; (2) the Chinese government paid more attention to control the pollution in the environment and enforced strict emission standards, which reduced the negative effect on the population health [35]. Moreover, one interesting finding is that the decrease of susceptible *S*(*t*) is beneficial to the decreasing trend of infected cases *I*(*t*); that is, if we can reduce the number of the susceptible subpopulation, the cases of measles will show a decreasing trend in the future (see Figure 5, blue lines). Thus, strengthening the coverage of measles vaccination and the environmental quality improvement are advantageous in controlling the outbreak of infectious diseases (e.g., measles).

## 4. Conclusion

Generally, humans are exposed to some kinds of infectious diseases because the diseases propagate through a polluted environment. Examples include measles spreading through air pollution, snail fever spreading through water pollution, and diarrhea spreading through food pollution. Understanding the transmission of an infectious disease is crucial to predict and prevent major outbreaks of an epidemic [17]. Thus, one of the fundamental questions for the dynamical models of infectious diseases is to find the conditions that identify whether an infectious disease will exhibit a periodic outbreak or not and determine the risk factors of pollution exposure for such an outbreak in the population. In this paper, we considered a stochastic SI epidemic model in a polluted environment and incorporated the effect of environmental fluctuations as well as pollution. First, we discussed the existence of the global positive solution to the model. The main result of this paper was to obtain the sufficient conditions of the nontrivial stochastic periodic solution (see Figure 2) and the boundary periodic solution of disease extinction (see Figure 4).

Compared to existing research, the main breakthrough of this paper is that we incorporated both environmental white noise as well as pollution into an SI epidemic model, which described two kinds of common phenomena in the transmission process of infectious diseases and explored the effects of environmental fluctuations (noise and pollution) on the dynamical behaviors of an epidemic. The numerical simulations based on the cases of measles showed the following:

- The environmental noise *σ* on *β* may play an important role in determining the epidemic pattern: (1) it follows from Theorems 8 and 12 that the large intensity of noise *σ* may adversely affect the existence of the stochastic periodic of model (7) and accelerate the extinction of infectious disease. Thus, the large intensity noise may help the control of periodic outbreak of infectious disease; (2) according to Figure 2, we can see that environmental fluctuations may be responsible for the variations in the seasonal outbreak pattern of a disease in a polluted environment.
- The pollution level (*r*_*i*0_*C*_0_^*∗*^) plays an important role in susceptible populations, in that it may reduce the number of susceptible population due to the effect of pollution; that is, the pollution causes serious harm to the susceptible population. Therefore, the pollution level may have adverse health effects on the susceptible exposed population, which is also be supported by the measles data (see Figure 5; the decreasing susceptible *S*(*t*) may be responsible for the decreasing tendency of *I*(*t*).) Moreover, by comparing the peak level of infective population with different average pollution levels *r*_*i*0_*C*_0_^*∗*^ (see Figure 2), we can see that the peak level of infective population increases with the levels of pollution.
- From an epidemiological viewpoint, our results may provide some theoretical evidence for controlling the infectious disease. For example, in the cases of measles, the strengthening coverage of the measles vaccination and environmental quality improvement are still effective prevention measures in a polluted environment. That is to say, the lesser the population falling within the scope of susceptible subpopulation, the less infected the patients. Therefore, the demographic characteristics of susceptible population may affect the periodic outbreaks of infectious disease. In addition, the pollution control is beneficial to population health, which is consistent with the environmental research results of hemorrhagic fever [37] and influenza [38].

However, this study also has several limitations:

- A key assumption of our model is that the pollution affects population dynamics with a linear dose-response function. Since the complicated mechanism of interaction between the pollution and population is still unclear, the dose-response parameter estimation is difficult, since the effect of pollution in vivo is not measurable for human patients. Thus, our model can not accurately describe this interaction, and the numerical simulations do not yet use the polluted data (such as PM2.5 or PM10) to check the effect of the pollution.
- The times series of pollution concentration presents significant variability [39]; however, our model has not included the variability of pollution.
- Some other issues also need to be considered in future, for example, the age-structured modeling [40], the impulse pollution input [41, 42], or the population with partial pollution tolerance [43].

## Figures and Tables

**Table 1:** Biological meanings of the parameters in model (1).

Parameters	Biological meanings	Unit
*γ*	The intrinsic growth rate in absence of the toxicant	t^−1^
*K*	Carrying capacity of the population in absence of the toxicant	Person
*β*	Probability of infection	-
*ξ*	The population natural death rate	t^−1^
*v*	The death rate caused by disease	t^−1^
*r*_10_	The dose-response rate due to uptake of pollution for the susceptible	t^−1^
*r*_20_	The dose-response rate due to uptake of pollution for the infected	t^−1^
*α*	The organisms net uptake rate of pollution from environment	t^−1^
*g*	The egestion rate of pollution in the organism (metabolism)	t^−1^
*m*	The depuration rate of pollution in the organism	t^−1^
*h*	The environmental pollution loss rate due to natural degradation	t^−1^
*u*(*t*)	The exogenous rate of pollutant input into the environment	t^−1^

**Table 2:** The values of the parameters in model (46).

Parameters	Mean value	Source
*γ*	0.01295	[30]
*β*	0.00495	Estimated
*ξ*	0.0067	[30]
*σ*	0.05	[30]
*K*	1.378 × 10^9^	[30]
*v*	0.00175	[31]
*r*_*i*0_, *i* = 1,2	0.0015	Estimated
*C*_0_^*∗*^	1.05	[1]

## Appendix

Proof of Lemma 2. It follows from the periodicity of *u*(*t*) that the solution *C*_*e*_^*∗*^(*t*) of *dC*_*e*_^*∗*^(*t*) = [−*hC*_*e*_^*∗*^(*t*) + *u*(*t*)]*dt* is a positive *θ*-periodic continuous function, that is,

(A.1) $$\begin{array}{c} C_{e}^{*}\left(\;t\;\right)=\frac{\int_{t}^{t+\theta }e^{h\left(s-t\right)}u\left(s\right)ds}{e^{h\theta }-1}. \end{array}$$

Combining with the third equation of model (4) can result in

(A.2) $$\begin{array}{c} C_{e}\left(\;t\;\right)-C_{e}^{*}\left(\;t\;\right)=\left(\;C_{e}\left(\;0\;\right)-C_{e}^{*}\left(\;0\;\right)\;\right)e^{-ht}, \end{array}$$

which satisfies *d*(*C*_*e*_(*t*) − *C*_*e*_^*∗*^(*t*)) = −*h*(*C*_*e*_(*t*) − *C*_*e*_^*∗*^(*t*))*dt*. Similarly, we can also get that the solution *C*_0_^*∗*^(*t*) of *dC*_0_^*∗*^(*t*) = [*αC*_*e*_^*∗*^(*t*)−(*g* + *m*)*C*_0_(*t*)]*dt* is a positive *θ*-periodic continuous function. According to the variation-of-constants formula, we have

(A.3) C0t−C0∗t=e−g+mtC00−C0∗0+α∫0tCes−Ce∗seg+msds=C00−C0∗0−αCe0−Ce∗0g+m−he−g+mt+αCe0−Ce∗0g+m−he−ht,

which satisfies *d*(*C*_0_(*t*) − *C*_0_^*∗*^(*t*)) = [*α*(*C*_*e*_(*t*) − *C*_*e*_^*∗*^(*t*))−(*g* + *m*)(*C*_0_(*t*) − *C*_0_^*∗*^(*t*))]*dt*. Thus, we can obtain

(A.4) ∫0tC0s−C0∗sds=1g+mC00−C0∗0−αCe0−Ce∗0g+m−h·1−e−g+mt+αCe0−Ce∗0g+m−h1−e−ht<+∞.

By virtue of the Barbălat Lemma [44] and (A.4), we obtain the required assertion.

Proof of Theorem 7. Adding the two equations of model (7) yields

(A.5) dSt+Itdt=γtSt1−St+ItKt−r10tC0∗tSt−ξSt−r20C0∗tIt−vtIt≤γuSt+It−γlSt+It2Ku,

which implies limsup_*t*→*∞*_(*S*(*t*) + *I*(*t*)) = *γ*^*u*^*K*^*u*^/*γ*^*l*^. Thus, the set

(A.6) $$\begin{array}{c} \Lambda =\left\{\;\left(\;S,I\;\right)\in R_{+}^{2},\;\;S+I\leq \frac{\gamma^{u}K^{u}}{\gamma^{l}}\;\right\} \end{array}$$

is a positively invariant set of system (7). Due to the coefficients of model (7) are local Lipschitz continuous. According to Theorem 3.15 in Mao [32], for any given initial value (5), there is a unique local saturated solution (*S*(*t*), *I*(*t*)) on *t* ∈ [0, *τ*_*e*_) where *τ*_*e*_ is the explosion time. To show this solution is global, we only need to show that *τ*_*e*_ = *∞* a.s. Since the initial value is positive and bounded, throughout this paper, let *m*_0_ be sufficiently large such that both *S*(0) and *I*(0) lie in the interval [*m*_0_^−1^, *m*_0_]. For each integer *m* ≥ *m*_0_, define the stopping time

(A.7) τm=inf⁡t∈0,τe:St∉1m,m  or  It∉1m,m,

and for the empty set define inf⁡*Ø* = *∞*. Then, *τ*_*m*_ is an increasing function in terms of *m* and *τ*_*∞*_ = lim_*m*→*∞*_⁡*τ*_*m*_ ≤ *τ*_*e*_ a.s. If we can show that *τ*_*∞*_ = *∞* a.s. then *τ*_*e*_ = *∞* a.s. and therefore (*S*(*t*), *I*(*t*)) ∈ *ℝ*_+_^2^ a.s. for all *t* ≥ 0. That is to say, to complete the proof all we need to show is that *τ*_*∞*_ = *∞* a.s. If this statement is false, then for any constant *T* > 0 there is an *ϵ* ∈ (0,1) such that *𝒫*{*τ*_*∞*_ ≤ *T*} > *ϵ*. Hence, there is an integer *m*_1_ ≥ *m*_0_ such that

(A.8) $$\begin{array}{c} P\left\{\;\tau_{m}\leq T\;\right\}\geq \epsilon ,\;\forall m\geq m_{1}. \end{array}$$

Define the *C*^2^ functional *U* : *ℝ*_+_^2^ → *ℝ*_+_:

(A.9) $$\begin{array}{c} U\left(\;S,I\;\right)=-\ln\left(\;\frac{\gamma^{l}S\left(t\right)}{\gamma^{u}K^{u}}\;\right)+I\left(\;t\;\right). \end{array}$$

By use of Itô's formula, we have

(A.10) dUSt,It=−γt+γtKtS+I+βtI+r10tC0∗t+ξt+βtSI−r20tC0∗t+vtI+12σ2tI2dt+σtI1+SdBt≤γuKlS+I+βuI+r10C0∗+ξu+βuSI+12σu2I2dt+σuI1+SdBt≤γuKuKl+r10C0∗+ξu+βuγuKuγl+βu+12σu2γuKuγl2dt+σuI1+SdBt=H1dt+σuI1+SdBt,

where

(A.11) H1=γuKuKl+r10C0∗+ξu+βuγuKuγl+βu+12σu2γuKuγl2<+∞.

Integrating both sides of (A.10) from 0 to *τ*_*m*_∧*t* = min⁡{*τ*_*m*_, *t*}, and then taking expectations, yields

(A.12) EUSτm ∧ t,Iτm ∧ t≤US0,I0+H1Eτm ∧ t.

Set *Ω*_*m*_ = {*τ*_*m*_ ≤ *T*} and it follows from (A.8) that *𝒫*{*Ω*_*m*_} ≥ *ϵ*. Note that, for every *w* ∈ {*τ*_*m*_ ≤ *T*}, there exists one of *S*(*τ*_*m*_, *w*) and *I*(*τ*_*m*_, *w*) equals either *m* or 1/*m*, and hence *U*(*S*(*τ*_*m*_, *w*), *I*(*τ*_*m*_, *w*)) is no less than

(A.13) C=min⁡−ln⁡γlmγuKu+m,−ln⁡γlmγuKu+1m,−ln⁡γlγuKum+m,−ln⁡γlγuKum+1m.

Consequently, it follows from (A.12) that

(A.14) Pτm≤TC≤E1τm≤TwUSτm,w,Iτm,w≤US0,I0+H1T,

where 1_{*τ*_*m*_≤*T*}_ is the indicator function of {*τ*_*m*_ ≤ *T*}. Letting *m* → *∞* gives lim_*m*→*∞*_*𝒫*(*τ*_*m*_ ≤ *T*) = 0, which contradicts with (A.8). So, *τ*_*e*_ = *∞*. Further, notice *T* > 0 is arbitrary. It then follows that *𝒫*(*τ*_*e*_ = *∞*) = 1. This completes the proof.
